# Frontotemporal dementia and parkinsonism linked to chromosome 17 (FTDP-17)

**DOI:** 10.1186/1750-1172-1-30

**Published:** 2006-08-09

**Authors:** Zbigniew K Wszolek, Yoshio Tsuboi, Bernardino Ghetti, Stuart Pickering-Brown, Yasuhiko Baba, William P Cheshire

**Affiliations:** 1 Department of Neurology, Mayo Clinic, FL, USA; 2 Fifth Department of Internal Medicine, Fukuoka University, Fukuoka, Japan; 3 Department of Pathology, Indiana University, Indianapolis, IN, USA; 4 Division of Laboratory and Regenerative Medicine, University of Manchester, Manchester, UK

## Abstract

Frontotemporal dementia and parkinsonism linked to chromosome 17 (FTDP-17) is an autosomal dominant neurodegenerative disorder, which has three cardinal features: behavioral and personality changes, cognitive impairment, and motor symptoms. FTDP-17 was defined during the International Consensus Conference in Ann Arbor, Michigan, in 1996. The prevalence and incidence remain unknown but FTDP-17 is an extremely rare condition. It is caused by mutations in the *tau *gene, which encodes a microtubule-binding protein. Over 100 families with 38 different mutations in the *tau *gene have been identified worldwide. The phenotype of FTDP-17 varies not only between families carrying different mutations but also between and within families carrying the same mutations. The pathogenetic mechanisms underlying the disorder are thought to be related to the altered proportion of tau isoforms or to the ability of tau to bind microtubules and to promote microtubule assembly. Definitive diagnosis of FTDP-17 requires a combination of characteristic clinical and pathological features and molecular genetic analysis. Genetic counseling should be offered to affected and at-risk individuals; for most subtypes, penetrance is incomplete. Currently, treatment for FTDP-17 is only symptomatic and supportive. The prognosis and rate of the disease's progression vary considerably among individual patients and genetic kindreds, ranging from life expectancies of several months to several years, and, in exceptional cases, as long as two decades.

## Disease name and synonyms

Frontotemporal dementia and parkinsonism linked to chromosome 17 (FTDP-17).

## Definition/diagnostic criteria

The term frontotemporal dementia and parkinsonism linked to chromosome 17 was defined during the International Consensus Conference in Ann Arbor, Michigan, in 1996 [1]. At the time, affected individuals with frontotemporal dementia and parkinsonism linked to the *wld *locus on chromosome 17 had been identified within 13 families. This syndrome is a familial disorder with autosomal dominant inheritance. The three major clinical features include behavioral disturbances, cognitive impairment, and parkinsonism. There are no strict diagnostic criteria for FTDP-17. Nevertheless, FTDP-17 should be considered in the differential diagnosis in the presence of one or more of the following [2]:

• Age of onset of neurological symptoms between the third and fifth decades;

• Progressive neuropsychiatric syndrome including personality and behavioral abnormalities and/or frontotemporal dementia;

• Parkinsonism-plus syndrome (bradykinesia, rigidity, postural instability, paucity of resting tremor, and poor or no response to dopaminergic therapy) frequently associated with falls and supranuclear gaze palsy and less commonly associated with apraxia, dystonia, and lateralization;

• Progressive speech difficulties from the onset of the illness;

• Seizure disorder poorly controlled by standard anticonvulsant therapy;

• Positive family history suggestive of autosomal dominant inheritance of a neurodegenerative disorder, even if there has been variability in clinical presentations.

## Epidemiology

**Figure 1:**
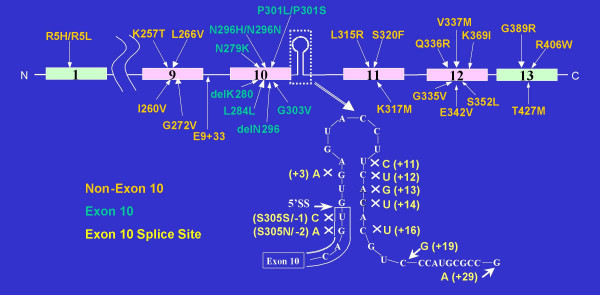
Schematic representation of the exons and introns of the tau gene localized on chromosome 17. The 38 mutations identified so far are presented.

FTDP-17 is an extremely rare condition. Its prevalence and incidence remain unknown. Over 100 families with FTDP-17 have been reported to date in numerous countries (USA, Great Britain, Japan, Netherlands, France, Canada, Australia, Italy, Germany, Israel, Ireland, Spain and Sweden). Some of these families share a common founder [3]. We estimate that there have been about 50–600 patients described, with fewer than 70 individuals still living. Molecular genetic studies have identified 38 unique mutations in these families (Figure 1). The most common mutations, accounting for approximately 60% of known cases, are P301L, N279K and a splice site mutation (exon 10 +16) [4].

## Etiology

Mutations in the *tau *gene account for the vast majority of FTDP-17 cases. Tau is a microtubule-binding protein abundant in neurons and glia. In neurons, it is predominantly expressed in axons. Tau binds to and stabilizes microtubules and promotes microtubule assembly. The majority of the currently known mutations in coding region occur within the microtubule-binding region of *tau *gene. Most known mutations in non-coding regions affect the splicing of exon 10 [4]. The pathogenetic mechanisms in FTDP-17 are thought to be related to the altered proportion of tau isoforms or to the ability of tau to bind microtubules and to promote microtubule assembly.

Mutations in *tau *gene associated with FTDP-17 fall into two broad mechanistic groups. One group contains coding mutations (missense and two deletions) that, in recombinant protein studies and in transfected cell assays, have been shown to disrupt the binding of tau to microtubules. In addition, the majority of these mutations have also been shown to accelerate the aggregation of recombinant tau in the presence of polyanions. Thus, overall, these mutations are predicted both to increase the proportion of tau that is unbound to microtubules and available for aggregation and also to increase directly the tendency of the unbound tau to form filaments. The second group of *tau *mutations appears to cause disease by disrupting the alternative splicing of exon 10 and hence the ratio of 4R: 3R *tau*. These mutations comprise a mixture of coding changes, within exon 10 (N279K, delK280, L284L, N296N/H, delN296, P301L/S, G303V, and S305S/N) and also intronic mutations close to the 5' splice site of exon 10 (at positions +3, +11, +12, +13, +14, +16, +19, and +29). All but three of these mutations have been demonstrated to increase the splicing-in of exon 10 and hence the proportion of 4R *tau*. The exceptions are delK280, +19, and, +29 mutations that show increased 3R: 4R ratio as compared with normal condition [5]. The +29 mutation has been detected in both affected individuals with the FTD phenotype and normal controls [6-8]. Therefore, it is still unclear whether this splicing mutation contributes to disease development.

### Families with FTD linked to chromosome 17q21 without tau mutations

Six FTDP-17 kindreds have been identified in which neither a *tau *mutation nor tau pathology has been detected, despite genetic linkage to the same region of chromosome 17q21 that contains the *tau *gene [7-12]. Their clinical phenotype is indistinguishable from that of cases with known *tau *mutations. The specific mutations and their clinical presentations are summarized in Table 1. Studies of this group of individuals provide valuable information for elucidating the etiology of FTDP-17, as it introduces the possibility that further mutations in the introns or regulatory regions of *tau*, or other causative genes near the *tau *locus may yet be discovered. For example, mutations in the intronic sequence adjacent to the stem loop structure in exon 10 have been identified that alter *tau *splicing to increase soluble 3R *tau*, leading to increased tau proteolysis and neuronal apoptosis without deposition of insoluble tau aggregates [13].

**Table 1:** Families with FTD linked to chromosome 17q21 without *tau *mutations.

Author	Froelich *et al*. [7]	Rosso *et al*. [8]	Lendon *et al*. [9]*	Rademakers *et al*. [10]	Kertesz *et al*. [11]	Bird *et al*. [12]^†^
	Karolinska	Dutch III	HDDD2	1083	Kertesz	Seattle B
Pedigree						
Origin	Sweden	Germany	USA	Netherlands	Canada	USA
No. of FA/G/AI	20/4/11	NA/4/32	>10,000/8/24	73/4/16	30/4/10	52/4/18
Mean ± SD age at onset, y	51 ± 3.6	61.2 ± 8.8	60.4 ± 0.5	64.9 ± 8.9	63.4 ± 12.5	54.7 ± 7.5
Mean ± SD disease duration, y	2.9 ± 0.8	8.6 ± 2.9	6.9 ± 2.2	6.6 ± 2.6	4.0 ± 2.7	10.1 ± 5.2
Clinical characteristics						
Initial sign	D	D/PC	D	D/PC	PC	PC (PS)
Dementia	+	+	+	+	+	+
Other features	Apraxia, dysphagia	LD	LD, EP, hemiparesis	LD	LD, dysphagia	Myoclonus
Predominant clinical phenotype	FTD	FTD	FTD (HDD)	FTD	FTD (Pick complex)	FTD
Pathologic findings	Severe frontal lobe degeneration with spongy changes and gliosis	Severe frontal lobe degeneration with neuronal loss and gliosis Neuronal loss in the hippo-campus	Severe frontal lobe degeneration with neuronal loss and gliosis Neuronal loss in the hippocampus	Frontotemporal degeneration with neuronal loss and gliosis	Frontotemporal lobe degeneration	Tau-positive NFT in the neocortex and limbic system
Ubiquitin-positive neuronal intranuclear inclusions	+	+	NR	+	+	NR
Max lod score for chromosome 17q21	2.68	3.46	3.68	5.51	1.68	1.11

+, present; AI, affected individual; D, dementia; EP, extrapyramidal signs; FA, family member; FTD, frontotemporal dementia; G, generation; HDD, hereditary dysphasic dementia; LD, language difficulties; NA, not available; NFT, neurofibrillary tangles; NR, not reported; PC, personality change; PS, psychiatric symptom.

*Data are from individuals with some form of dementia in recent generations of the family

Extensive ubiquitin-positive, tau-negative, cytoplasmic and intranuclear neuronal inclusions have been observed in layer II of the cerebral cortex and in the dentate gyrus of the hippocampus in three of the kindreds lacking a *tau *mutation [14,15]. The intranuclear inclusions are unlikely to result from a trinucleotide repeat expansion mutation in that they failed to stain when exposed to an antibody that recognizes proteins with polyglutamine tracts [7]. Descriptions of similar intranuclear inclusions in patients with FTD and motor neuron disease [14,15] suggest that they may turn out to be specific for chromosome 17-linked FTD without *tau *mutations.

## Clinical description

### Symptoms and signs

The onset of FTDP-17 is usually insidious. Affected individuals who have reached the fully developed stage of the disease present with a constellation of signs including at least two of the three cardinal features of FTDP-17.

• Behavioral and personality disturbances;

• Cognitive deficits;

• Motor dysfunction (typically signs of parkinsonism-plus syndrome).

Clinical features of FTDP-17 vary considerably among affected individuals, regardless of whether they inherit the same or different mutations. Even members within a family, for example, can vary in their clinical presentation. The specific mutations and their characteristic clinical presentations are summarized in Table 2.

**Table 2:** Phenotypes of specific *tau *gene mutations seen in FTDP-17 patients.

	Exon								
Clinical feature	1	9	11	12	13	10	Intron 10		
Average age at onset, y									
≤30				S352L		P301S			
				G335V					
31–40		L266V	S320F		G389R	delN296			
						S305N			
						G303V			
41–50		G272V	K317M	E342V		N279K	+3	+11	+14
		K257T				P301L	+16		
>50	R5H	I260V	L315R	V337M	R406W	del280K	+12	+13	+19
	R5L			Q336R	T427M	L284L	+29		
				K369I		N296H			
						S305S			
						N296N			
Average duration, y									
≤5	R5H	L266V	L315R	S352L	G389R	del280K	+29		
	R5L	K257T				delN296			
						N296H			
						S305N			
						G303V			
6–10		G272V I260V	K317M	E342V Q336R	T427M	N279K P301L	+11	+12	
				K369I		S305S			
				G335V		N296N			
						L284L			
						P301S			
11–15			S320F	V337M			+3	+14	+16
>15					R406W				
First sign									
Parkinsonism	R5L		K317M			N279K	+3	+11	
						P301L			
						delN296			
						S305S			
						N296N			
						G303V			
Dementia	R5H	K257T	S320F	Q336R	R406W	L284L	+3	+12	+29
			L315R			delN296			
Personality change		G272V	L315R	V337M	T427M	P301L	+12	+14	+16
		I260V		E342V		N296H	+19	+29	
				K369I		S305S			
				G335V		S305N			
Parkinsonism									
Early-prominent			K317M			N279K	+11		
						delN296			
						S305S			
						N296N			
						G303V			
Late-prominent						P301S	+3	+12	+14
						N296H	+16	+29	
Rare-minimal	R5L	G272V		S352L		P301L			
						S305N			
Dementia									
Early-prominent		L266V	S320F	Q336R	R406W	delN296	+12	+29	
		K257T	L315R	G335V	T427M	S305S			
		I260V							
Late-prominent		G272V		V337M		del280K	+3	+11	+13
				K369I		L284L			
						P301L			
						P301S			
						S305N			
Rare-minimal			K317M			N279K G303V			
Personality change									
Early-prominent		L266V	L315R	V337M	R406W	del280K	+12	+14	+16
		G272V		K369I	T427M	L284L	+19	+29	
		I260V		G335V		P301L			
		K257T				P301S			
						S305S			
						S305N			
Other clinical features									
Language s		G272V	S320F	Q336R	G389R	N279K	+14	+16	+29
difficultie		K257T	L315R		T427M	L284L			
		I260V	K317M			N296H			
						P301L			
						S305S			
						N296N			
						G303V			
Late mutism			L315R	V337M	R406W	N279K	+16	+29	
					T427M	del280K			
						N296H			
						P301S			
						S305N			
Eye movement						N279K	+3		
abnormalities						N296H			
						P301S			
						delN296			
						S305S			
						N296N			
						G303V			
Epilepsy				S352L		P301S			
Myoclonus				V337M		N279K	+11		
				S352L		P301S			
Pyramidal signs						N279K	+3	+12	
						P301S			
						N296N			
						G303V			
						S305S			
Amyotrophy			K317M			P301L N296N	+14		

The behavioral and personality abnormalities can include disinhibition, apathy, defective judgment, compulsive behavior, hyper-religiosity, neglect of personal hygiene, alcoholism, illicit drug addiction, verbal and physical aggressiveness, family abuse, and other manifestations. While cognitive disturbances occur, memory, orientation, and visuospatial function are relatively preserved during early stages of the disease. Progressive speech difficulties with non-fluent aphasia and disorders of executive functions can be seen initially. Subsequently, memory, orientation, and visuospatial functions progressively deteriorate, while echolalia, palilalia, verbal and vocal perseverations develop. Finally, progressive dementia and mutism occur. Motor signs are also prominent. Parkinsonism can be the first manifestation of the disease, and in this regard it is important to note that some FTDP-17 patients were initially misdiagnosed as having Parkinson's disease or sporadic progressive supranuclear palsy. In some families, however, the parkinsonism occurs late in the course of the illness or not at all. The parkinsonism in FTDP-17 is characterized by rather symmetrical bradykinesia, postural instability, rigidity affecting equally axial and appendicular musculature, (usually) absence of resting tremor, and poor or no responsiveness to levodopa therapy. Other motor disturbances seen in FTDP-17 include dystonia unrelated to medications, supranuclear gaze palsy, upper and lower motor neuron dysfunction, myoclonus, postural and action tremors, eyelid opening and closing apraxia, dysphagia, and dysarthria.

### Phenotype-genotype correlations

It is still very difficult to perform precise phenotype/genotype correlations in FTDP-17 since the clinical information available for some families is not detailed enough in or not accessible at all. Nevertheless, some patterns have emerged. The families with FTDP-17 fall into two major groups [16]:

• dementia predominant phenotype;

• parkinsonism-plus predominant phenotype.

The dementia predominant phenotype is more common and is usually seen in families with mutations in exons 1, 9, 11, 12, 13 and in exon 10.

The parkinsonism-plus predominant phenotype is usually seen in families with intronic and exonic mutations affecting exon 10 and leading to the selective overproduction of 4R *tau *isoforms. These categorizations should be viewed cautiously until more clinical and pathologic data become available.

## Diagnostic methods

Characteristic clinical and pathological features of FTDP-17 coupled with a molecular genetic analysis of the *tau *gene are essential steps for a diagnosis.

Imaging studies such as computerized tomography (CT) and magnetic resonance imaging (MRI) can assist in establishing a diagnosis, mainly by excluding other diagnostic possibilities such as the presence of a brain tumor, abscess, multi-infarct state, or hydrocephalus.

### Imaging studies

**Figure 2:**
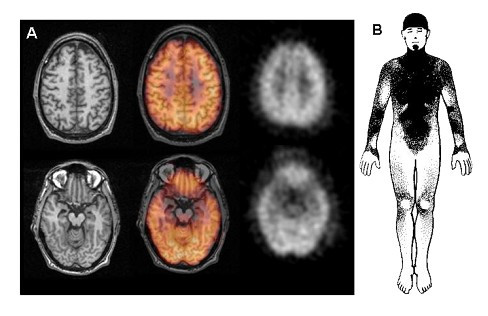
A) Head MRI (left panels) demonstrates cortical atrophy in frontal and temporal lobes. Positron emission tomography (PET) with 2-deoxy-2-fluoro- [^18^F]-D-glucose (FDG) PET (right panels) demonstrates hypometabolism in the same cortical regions. Middle panels show co-registration of MRI and FDG PET studies. B) Thermoregulatory sweating tests in the same patient with PPND. Shaded areas represent sweating over the anterior body surface. Distal anhidrosis is seen.

CT and MRI of the head usually demonstrate some dilation of the ventricular system and frontal, temporal, and parietal cortical atrophy (Figure 2A) [17-22]. MRI T2-weighted images may show accumulation of paramagnetic substances (iron) in mesencephalic nuclei [19]. In some kindreds, asymmetrical cortical atrophy is present. Functional imaging studies such as single photon emission computerized tomography (SPECT) and positron emission tomography (PET) also demonstrate significant abnormalities. PET with 2-deoxy-2-fluoro- [^18^F]-D-glucose (FDG) usually shows reduced frontal-parietal-temporal uptake patterns similar to those seen in sporadic frontotemporal dementia (FTD). PET with [^18^F]-fluoro-L-dopa (6FD) and [^11^C]-raclopride tracers reveals uptake abnormalities distinct from those seen in PD, in which the putamen is affected more than the caudate nucleus [23].

### Laboratory findings

The routine serum, urine, cerebrospinal fluid and other body fluid studies are usually negative. Electroencephalography (EEG) findings are normal early in the disease process and progress to diffuse slowing as the disease advances, although slowing can sometimes be seen earlier in the course of the FTDP-17 illness [24]. In sporadic FTD, the slowing of background rhythms usually occurs late in the course of the illness. In individuals with the P301S mutation, EEG demonstrates sharp waves, spikes and epileptiform discharges [25]. Nerve conduction studies are normal. Electromyography may show neurogenic patterns related to lower motor neuron dysfunction [26]. Evoked potential studies are usually normal. Autonomic testing may show sudomotor impairment but not orthostatic hypotension (Figure 2B) [27]. There is very little information available on sleep studies in FTDP-17 kindreds. In a clinicopathological study of an affected patient with G389R mutation, a relationship between the reduced delta sleep and the decreased cortical and subcortical connectivity was reported [28]. Neuropsychological evaluation is of paramount significance in determining the severity and extent of cognitive and behavioral dysfunction in this disorder.

## Differential diagnosis

Clinically, FTDP-17 may mimic several neurodegenerative diseases. The differential diagnosis of FTDP-17 includes disorders with initial signs such as behavioral and personality abnormalities, parkinsonism, lower motor neuron dysfunction or cognitive impairments. In the absence of a positive family history or molecular genetic data, FTDP-17 is most frequently confused with Pick's disease, sporadic progressive supranuclear palsy (PSP) or corticobasal degeneration (CBD). Other familial frontotemporal dementias, parkinson-plus syndromes, dementia with Lewy bodies (DLB), Parkinson's disease (PD), multiple system atrophy (MSA) should be excluded. A neuropathologic examination coupled with molecular genetic analysis of the *tau *gene are essential steps towards distinguishing FTDP-17 from other neurodegenerative diseases associated with tau deposition. The pathologic analysis should include immunohistochemical studies using multiple anti-tau antibodies.

## Genetic counseling

Affected individuals should be counseled regarding the estimated probability of passing the genetic bases for their illness on to their offspring. As FTDP-17 is an autosomal dominantly inherited condition, each offspring of an affected individual will carry a 50% risk of inheriting the abnormal gene. Some of these mutations can be detected through genetic testing. The individual who inherits a mutation will not necessarily develop the same clinical syndrome as the parent, because penetrance may be incomplete, neurologic manifestations vary greatly even within families, or the individual might die from unrelated causes. In the case of the pallido-ponto-nigral degeneration (PPND) subtype of FTDP-17, however, penetrance is complete and virtually all individuals who inherit the mutation will become symptomatic during middle age.

At-risk individuals may sometimes choose to adopt rather than bear their own children. When faced with the option of genetic testing, some people prefer the greater certainty that a genetic diagnosis may afford when planning life decisions, while others find not knowing to be a lesser emotional burden as well as a lesser risk of being denied healthcare should their insurance carrier learn the test result. In either case the patient's choice should be respected.

Inquiring into the family history can also identify relatives who may be at risk of having inherited the genetic basis for FTDP-17 and who thus may be presymptomatic.

Clinical genetic testing of affected and presymptomatic individuals is commercially available for some mutations. However, there tends to be little interest among at-risk family members to undergo clinical genetic testing [29].

## Antenatal diagnosis

Once a genetic basis for a subtype of FTDP-17 is discovered and testing for that gene becomes commercially available, it becomes possible, in principle, to identify that gene antenatally by sampling amniotic fluid or by preimplantation genetic diagnosis. Genetic counseling should accompany antenatal genetic diagnosis.

## Management including treatment

Currently, a curative treatment for FTDP-17 does not exist, and most patients respond poorly, if at all, to levodopa. Palliative and symptomatic interventions are the mainstay of treatment. Physical therapy can be helpful to preserve some measure of mobility and independence in activities of daily living and to reduce the risk of falling. Stool softening agents are recommended for constipation. Speech therapy, swallowing assessment, and switching to a softer diet may be helpful for dysphagia. Anticholinergics may be helpful for sialorrhea or urinary frequency but carry the risk of aggravating mental confusion. Clozapine or quetiapine may be helpful for psychosis not explained by systemic infection or responsive to medication withdrawal. Antidepressant medications may be indicated for concurrent depression. Bed-bound patients will require frequent repositioning or air flow mattresses. Patients should be encouraged to discuss their treatment preferences with their families and their physicians and to indicate their choices by advance directives.

## Prognosis

The prognosis varies considerably. Most patients with FTDP-17 will experience several months to one or two years of moderate impairment followed by an unremitting downward course of physical and cognitive decline. The rate of progression varies greatly among individual patients and genetic kindreds, ranging from life expectancies of several months to several years, and, in exceptional cases, as long as two decades. In addition to the clinical features described above, in the late stages of illness these patients often develop secondary medical problems associated with immobility including falling injuries and respiratory and urinary tract infections.

## Unresolved questions

It is hoped that further research will identify the genetic modifiers and environmental factors that influence the variable penetrance and clinical expression of FTDP-17. The development of transgenic mice may provide an opportunity to test novel therapeutic agents in the near future.
